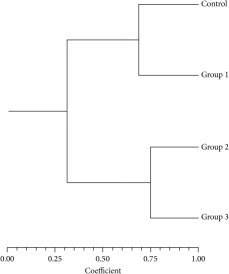# RAPD Profiling, DNA Fragmentation, and Histomorphometric Examination in Brains of Wistar Rats Exposed to Indoor 2.5 Ghz Wi-Fi Devices Radiation

**DOI:** 10.1155/2017/8653286

**Published:** 2017-08-20

**Authors:** A. O. Ibitayo, O. B. Afolabi, A. J. Akinyemi, T. I. Ojiezeh, K. O. Adekoya, O. O. Ojewunmi

**Affiliations:** ^1^Biological Sciences Department, Afe-Babalola University, Ado-Ekiti, Ekiti State, Nigeria; ^2^Biochemistry Department, Afe-Babalola University, Ado-Ekiti, Ekiti State, Nigeria; ^3^Medical Laboratory Sciences Department, Afe-Babalola University, Ado-Ekiti, Ekiti State, Nigeria; ^4^Cell Biology and Genetics Department, University of Lagos, Akoka, Lagos State, Nigeria; ^5^DNA Laboratory, National Sickle Cell Centre, Idi Araba, Lagos State, Nigeria

## Abstract

The advent of Wi-Fi connected high technology devices in executing day-to-day activities is fast evolving especially in developing countries of the world and hence the need to assess its safety among others. The present study was conducted to investigate the injurious effect of radiofrequency emissions from installed Wi-Fi devices in brains of young male rats. Animals were divided into four equal groups; group 1 served as control while groups 2, 3, and 4 were exposed to 2.5 Ghz at intervals of 30, 45, and 60 consecutive days with free access to food and water ad libitum. Alterations in harvested brain tissues were confirmed by histopathological analyses which showed vascular congestion and DNA damage in the brain was assayed using agarose gel electrophoresis. Histomorphometry analyses of their brain tissues showed perivascular congestion and tissue damage as well.

## 1. Introduction 

Radiofrequency electromagnetic radiation (RF-EMR) emission rates are fast increasing among developing countries over the last decade. This can be traced to the perceived need and use of Internet services in executing daily activities at home, work, school, and so forth [1]. Technological advancement necessitated the use of Wi-Fi connected devices in communications, research, and many other spheres of life and speeding up of day-to-day activities [2, 3]. The various sources of RF-EMF emission include devices such as phones and laptops. However, a great debate exists about the possible damage the radiofrequency electromagnetic radiation (RF-EMR) emitted by mobile phones exerts on living systems which includes damage to brain cells, impairment of metabolic activities in tissues, and ultimately, damage to the hereditary materials DNA, which serves as the underlying molecular chaperon of diverse metabolic activities that takes place in living systems [4–6]. Health hazards from increased RF-EMF exposure has been recognized to cause a wide range of biochemical and physiological dysfunctions particularly in the brains of young ones [7]. Moreover, the long-term RF-EMF exposure generates reactive oxygen species and different free radicals [8]. Weak fields may speed up electron sharing process and thereafter weaken the H-bond of cellular macromolecules. This phenomenon could describe the mechanism of transcription and protein translation, which has been observed after RF-EMF long-term exposure [9]. On the other hand, the total energy of weak EM-fields is not directly enough to break a chemical bond in the genetic material [10]. Consequently, it could be deduced indirectly that genotoxicity is arbitrated by indirect mechanisms and afterward the generation of reactive radicals (ROS) or a disruption to DNA-repair processes [11, 12]. Also, RF-EMF exposure can induce alteration in plasma membrane potential and calcium efflux with resultant calcium diminution which leads to decrease in the activity of protein kinase C thereby promoting the alteration in many enzymes, ion pumps, channels, and proteins as well as inducing apoptosis [13]. Several studies confirmed that exposure to electromagnetic fields may increase the incidence of cancer and DNA damage of sperm and brain cells [14]. Other reports indicated deleterious hematopoietic indices, gastrointestinal, reproductive effects and so forth [15–17]. There are also experimental proofs of RF fields effects on human physiology and behavior at field strengths found in the home or environment [18].

## 2. Materials and Methods 

### 2.1. Animals and Experimental Design

Sixteen adolescent Wistar albino male rats weighing 80–120 g obtained from the animal house of Faculty of Science, Afe-Babalola University, Nigeria, were selected and used for the study. They were maintained for two weeks as acclimatization period under standard laboratory conditions with free access to food and water provided ad libitum and subjected to natural light for 12 hours and dark for 12 hours' cycles. After the period of acclimatization, rats were divided randomly into four groups, 4 animals in each group. The animals were pair-housed in groups of four in steel mesh cages and placed about 10 cm away from installed devices that emitted RF-EMF. The control group (*n* = 4) was kept in a steel mesh cages with a glass barrier separating the control from the experimental animals. The radiation exposure on the animals was varied from acute (30 days), subchronic (45 days), and chronic (60 days) exposure. Upon arrival, animals were weighed and handled. Ad libitum food (pelletized growers mash obtained from ABUAD farm) was weighed daily and given in equal proportions to the four groups. Distilled water was provided in stainless steel containers for the duration of the experiment.

### 2.2. Tissue Preparation

At the end of exposure, rats were fasted for 12 hours before being anesthetized and sacrificed by cervical dislocation. They were decapitated and their brains were immediately harvested and washed using chilled saline solution. The harvested brains of the animals from each group were minced using a homogenizer and separately homogenized (20% w/v) in ice cold Tris-HCL (0.01 M, pH 7.4) buffer.

### 2.3. Histomorphometry Analysis

Specimens of brain tissues were immediately fixed in 10% formal saline, treated with conventional grade of alcohol and xylol, embedded in paraffin, and sectioned at 4–6 *μ* thickness. The sections were stained with haematoxylin and eosin (H&E) stain for studying the histomorphometry changes.

### 2.4. Random Amplified Polymorphic DNA (RAPD) Technique

#### 2.4.1. Extraction of DNA

DNA was extracted from brains of the four groups following the method described by Bardakci and Skibinski [19].

#### 2.4.2. Polymerase Chain Reaction (PCR) Primers

In this study, ten-base long oligonucleotides primers were used to initiate the PCR amplifications. Primers were randomly selected based on GC content and annealing temperature for RAPD-PCR amplification (Table 1).

#### 2.4.3. PCR Amplification and Agarose Gel Electrophoresis

PCR amplifications were performed as described by Williams et al. 1993 [20] using the isolated DNA from one sample of each group.

#### 2.4.4. Agarose Gel Electrophoresis

The amplified DNA fragments were separated on 1.5% agarose gel and stained with ethidium bromide. DNA ladder 1 kb plus ladder from Thermos Scientific was used in this study as marker for amplified pattern. The amplified pattern was visualized and photographed by gel documentation system.

### 2.5. Statistical Analysis

The data entry was done into a binary data matrix as discrete variables and was analyzed according to Steel and Torrie [21]. Statistical significance of the difference in values of control and treated animals was calculated at 5% significance level. Data of the present study were statistically analyzed by using Duncan's multiple range test (SAS Analytics). All RAPD profiles were analyzed using stat program which showed the similarity between the amplified PCR products. The best amplified PCR product of each group was selected to compare between the genetic levels.

## 3. Results

### 3.1. DNA Fragmentation and Genetic Analysis Using RAPD-PCR

**Figure 1:**
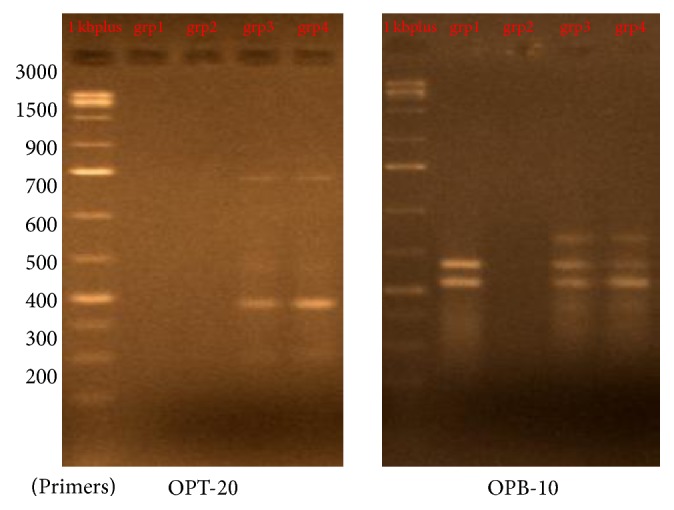
Figure 1 shows the RAPD profile of group 1, control (no radiofrequency exposure), group 2 (30-day RF exposure), group 3 (45-day RF exposure), and group 4 (60-day RF exposure) animals using OPH-20 and OPB-10 primer.

**Figure 2:**
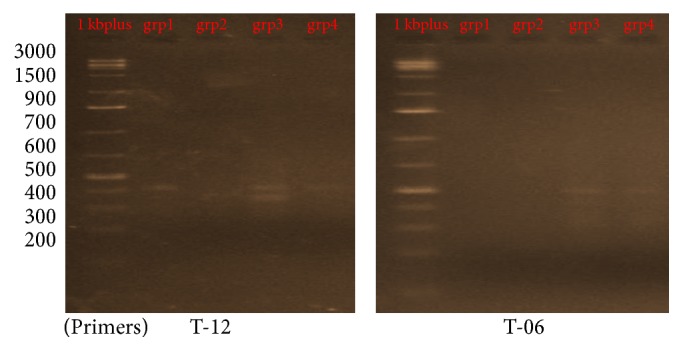
RAPD profile of group 1, control (no radiofrequency exposure), group 2 (4-week RF exposure), group 3 (6-week RF exposure), and group 4 (8-week RF exposure) animals using T-12 and T-06 primers.

**Figure 3:**
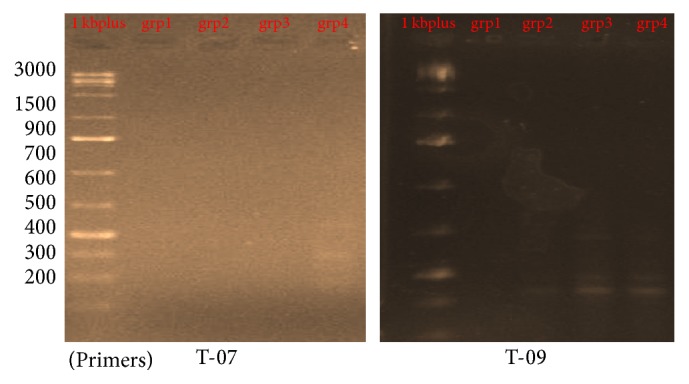
RAPD profile of group 1, control (no radiofrequency exposure), group 2 (4-week RF exposure), group 3 (6-week RF exposure), and group 4 (8-week RF exposure) animals using T-07 and T-09 primers.

Six of 10-mer primers were used for investigating the significant changes of the DNA isolated from brain tissues which was loaded on a 1.5% agarose gel. Two out of the six 10-mer primers (OPT – 20 and OPB – 10) produced clear, sharp, monomorphic, and polymorphic bands as seen in Figure 1. Primers 1 and 4 (OPT 06 and OPT 12) gave band patterns of almost the same profile between the four as seen in Figure 2 although some bands were lost. In contrast, primer 2 and primer 3 (OPT 07 and OPH 09) were least informative and they produced mostly undistinguishable banding profiles between the amplified samples of each group after RAPD assays as shown in Figure 3. The amplified fragments of PCR products were summarized as in Table 2. Primers 1 and 6 produced almost similar RAPD fingerprints for control group (group 1) while primers 1, 4, and 6 detected some changes in brain DNA of subchronic (group 3 for 45 days) and chronic (group 4 for 60 days) group showing similar band sizes. Primers 2 and 3 produced almost similar RAPD fingerprints for subchronic (group 3 for 45 days) and chronic (group 4 for 60 days) group although some bands were lost. The RAPD products were scored as present (1) or absent (0) for each primer-genotype combination. The results of RAPD patterns of the 6 primers were summarized as in Table 2. Sixteen bands were amplified and scored where 4 were polymorphic. Jaccard's coefficient of similarity was measured and a dendrogram constructed based on similarity coefficients which was generated by using unweighted pair group method with arithmetic mean (UPGMA). The best amplified PCR was selected from each group to compare between them using stat software. The analysis of the results described the similarity between different samples of brain tissues. The similarity of control (group 1) and group 2 (acute exposure) was about 61% and 71%, respectively, compared to group 3 and 4. The variations of the RAPD profiles were compared between the control and Wi-Fi-exposed groups.

**Figure 4:**
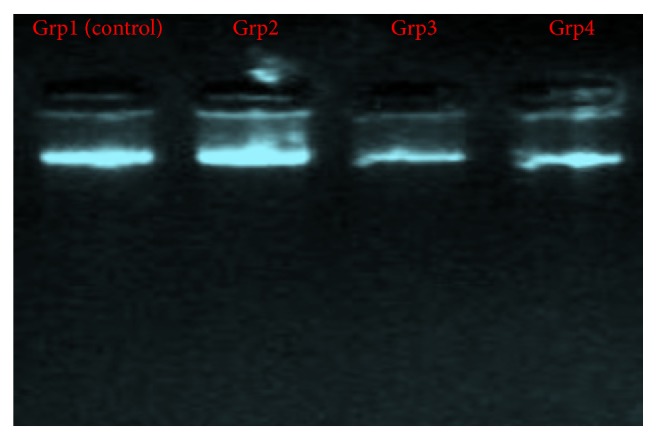
DNA fragmentation pattern using agarose gel electrophoresis.

We also observed a progressive DNA fragmentation in the band pattern as shown in Figure 4 using agarose gel electrophoresis where the DNA from the brains of the control rats (group 1) and group 2 (30-day RF exposure spectrum) showed similar fragmentation pattern and larger band sizes while the bands of groups 3 (45-day RF exposure spectrum) and 4 (60-day RF exposure spectrum) indicated DNA damage and fragmented band sizes signifying apoptosis from subchronic and chronic Wi-Fi exposed groups.

### 3.2. Histomorphometric Examinations

**Figure 5:**
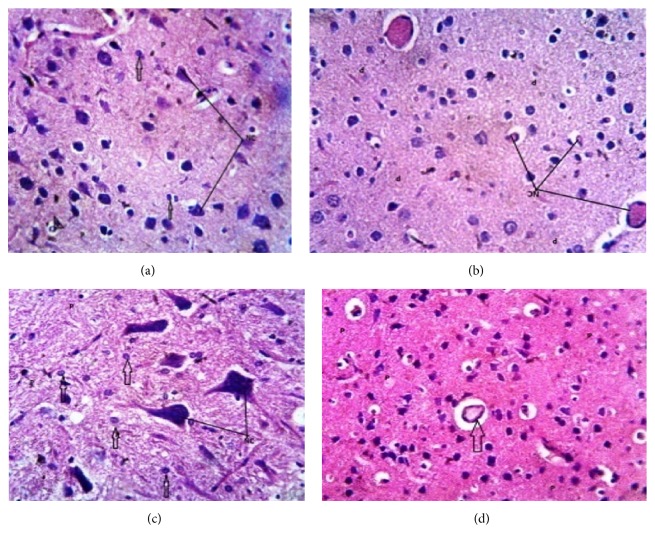
Paraffin sections stained by haematoxylin and eosin (H&E) ×400 for histomorphometry examination of brain (cerebrum) tissues of rats as follows. (a) Photomicrograph of control group shows normal architecture (H); section of the brain is composed of nerve cells (NC) and glia cells (arrow) and blood vessel (arrow head) disposed within the brain parenchyma. Section is free from collections and inflammatory cells infiltrations. (b) Photomicrograph of group 2 (30-day RF exposure) shows section of the brain with vascular congestion (L); the parenchyma is free from collection. (c) Photomicrograph of group 3 (45-day RF exposure) shows sections-nerve cells (NC) and neuroglia (arrow) appear as in control. (d) Photomicrograph of group 4 (chronic exposure) shows section of the brain with vascular congestion (L); the parenchyma is free from collection.

The histomorphometric studies of the groups are shown in Figure 5.

## 4. Discussion 

Radiation has been speculated to be an environmental pollutant and its toxicity has also been associated with health problems. The brain is the closest organ in the body to the ear when calls are received or placed. Hence, it is one of the target organs affected by radiation exposure owing to its fluidity and ions retention especially in young children [7, 22]. This accounts for the restrictions and recommendations on the bandwidth rate and types of cell phones that can be used by children in some countries. Data shown in Table 2 demonstrated that subchronic and chronic exposure to Wi-Fi radiation indicated DNA damage in their brain tissues while acute exposure revealed also relative effects which were not as severe as the longer exposure periods. Also, the PCR product using selected RAPD-PCR marker assisted analysis gave an evidence of DNA damage and mutations which was reflected in their RAPD profiles such as disappearance of bands and appearance of new PCR products which occurred in the profiles generated by rats exposed to Wi-Fi radiation. RAPD-PCR was used here to analyze induced DNA damage and changes in band patterns observed in DNA “fingerprint” analyses which reflects DNA alterations from single base changes (point mutations) to complex chromosomal rearrangements. Furthermore, random amplified polymorphism of DNA (RAPD) showed distinct differences in animal groups exposed for acute (30 days), subchronic (45 days), and chronic exposure periods. Also, gel electrophoresis of extracted DNA samples from these groups revealed a dose-dependent (based on exposure duration) deleterious effect of Wi-Fi radiation on the brain tissues and cells as shown in Figure 4. In addition to this, Figures 5(b) and 5(d) showed that Wi-Fi radiation exposure induced severe histopathological alterations in the acute and chronically exposed animals when compared to the control animals where there was vascular congestion and abnormal architecture which is indicative of oxidative stress and neuroinflammation and this is in consonance with the findings of [12, 18, 22], although radiation effects as indicated in animals from the subchronic group did not really exhibit severe histopathological changes as being observed in the acute and chronically exposed groups.

## 5. Conclusion 

In this study, the effect of Wi-Fi radiation exposure as a threat to brain health was studied using genomic analysis and histopathological study which showed the high risk of its genotoxicity especially in prolonged exposure spectrum through the findings from this study. The genomic analysis confirmed DNA damage due to Wi-Fi radiation toxicity and DNA damage effect which was seen through the RAPD profiles of animals from the exposed groups. The histopathological analyses also confirmed significant deleterious alterations in the brain tissues of Wi-Fi-exposed animals. Hence, the need to exhibit caution in handling smart devices that are used from day to day is fast becoming a threat to human health and wellness.

## Figures and Tables

**Table 1:** PCR primers used in RAPD-PCR.

1	OPT – 06	- 5′ – CAA	GGG	CAG	A – 3′
2	OPT – 07	- 5′ – GGC	AGG	CTG	T – 3′
3	OPH – 09	- 5′ – TGT	AGC	TGG	G – 3′
4	OPT – 12	5′ – GCG	TGT	CTA	G – 3′
5	OPT – 20	5′ – GAC	CAA	TGC	C – 3′
6	OPB – 10	5′ – CTG	CTG	GGA	C – 3′

**Table 2:** 

Primer	Number of amplified DNA fragment	Number of polymorphic DNA fragment	% polymorphism	% indexing
OPH-09	3	1	33.3	
OPT-06	2	—	—	
OPT-07	3	3	100	
OPB-10	3	—	—	
OPT-12	1	—	—	
OPT-20	4	—	—	

*Total*	16	4	—	25